# Molecular Basis for Mambalgin-2 Interaction with Heterotrimeric α-ENaC/ASIC1a/γ-ENaC Channels in Cancer Cells

**DOI:** 10.3390/toxins15100612

**Published:** 2023-10-13

**Authors:** Ekaterina N. Lyukmanova, Maxim M. Zaigraev, Dmitrii S. Kulbatskii, Aizek B. Isaev, Ilya D. Kukushkin, Maxim L. Bychkov, Mikhail A. Shulepko, Anton O. Chugunov, Mikhail P. Kirpichnikov

**Affiliations:** 1 Faculty of Biology, MSU-BIT Shenzhen University, Shenzhen 518172, China; mikhailshulepko@yandex.ru; 2 Shemyakin-Ovchinnikov Institute of Bioorganic Chemistry, Russian Academy of Sciences, Miklukho-Maklaya 16/10, Moscow 117997, Russia; maximzaigraev@yandex.ru (M.M.Z.); d.kulbatskiy@gmail.com (D.S.K.); isaev.aizek@gmail.com (A.B.I.); ilya23kuku@gmail.com (I.D.K.); maksim.bychkov@gmail.com (M.L.B.); batch2k@yandex.ru (A.O.C.); kirpichnikov@inbox.ru (M.P.K.); 3 Phystech School of Biological and Medical Physics, Moscow Institute of Physics and Technology (National Research University), Institutsky Lane 9, Dolgoprudny, Moscow 141701, Russia; 4 Interdisciplinary Scientific and Educational School of Moscow University «Molecular Technologies of the Living Systems and Synthetic Biology», Faculty of Biology, Lomonosov Moscow State University, Leninskie Gory, Moscow 119234, Russia

**Keywords:** three-finger toxins, acid-sensing ion channels, cancer, DEG/ENaC, ASIC1a, α-ENaC, γ-ENaC, mambalgin-2

## Abstract

Cancer progression is characterized by microenvironmental acidification. Tumor cells adapt to low environmental pH by activating acid-sensing trimeric ion channels of the DEG/ENaC family. The α-ENaC/ASIC1a/γ-ENaC heterotrimeric channel is a tumor-specific acid-sensing channel, and its targeting can be considered a new strategy for cancer therapy. Mambalgin-2 from the *Dendroaspis polylepis* venom inhibits the α-ENaC/ASIC1a/γ-ENaC heterotrimer more effectively than the homotrimeric ASIC1a channel, initially proposed as the target of mambalgin-2. Although the molecular basis of such mambalgin selectivity remained unclear. Here, we built the models of the complexes of mambalgin-2 with the α-ENaC/ASIC1a/γ-ENaC and ASIC1a channels, performed MD and predicted the difference in the binding modes. The importance of the ‘head’ loop region of mambalgin-2 for the interaction with the hetero-, but not with the homotrimeric channel was confirmed by site-directed mutagenesis and electrophysiology. A new mode of allosteric regulation of the ENaC channels by linking the thumb domain of the ASIC1a subunit with the palm domain of the γ-ENaC subunit was proposed. The data obtained provide new insights into the regulation of various types of acid-sensing ion channels and the development of new strategies for cancer treatment.

## 1. Introduction

The intensive metabolism is characteristic of tumors. Due to high cell density and insufficient oxygenation, metabolic pathways are activated in tumor cells, leading to the formation of acidic products that are removed from tumor cells to maintain a neutral intracellular pH [1,2,3]. This decreases the pH of the tumor microenvironment from ~7.4 to ~5.5–6.5 [4,5,6,7,8]. Tumor cells adapt to low environmental pH by activating pH sensors such as proton-sensitive cation channels of the degenerin/epithelial sodium channel (DEG/ENaC) family, which are trimers consisting of homologous subunits ASIC1a, ASIC1b, ASIC2a, ASIC2b, ASIC3, ASIC4, and α–δ ENaC [9,10,11,12,13]. The implication of these pH sensors in maintaining different (gastric, breast, hepatic, and glioblastoma) tumor cell growth is well documented [14,15,16,17,18,19]. The subunits that form these channels determine their pH sensitivity and pharmacological properties [20]. The most sensitive to a pH drop are the ASIC1a channels, which are expressed in the central nervous system and mediate synaptic plasticity and pain perception. Channels formed by ASIC1a subunits are expressed in non-neuronal cells, where they regulate gas exchange, the secretion of antibodies by lymphocytes, and the differentiation of keratinocytes in the skin [11]. ASIC1a channels are activated by a pH drop to ~6.4–6.6 and provide an entry of sodium ions into the cell. The inward sodium current regulates important cellular functions such as calcium homeostasis, volume and shape maintenance, activity of mitogenic signaling pathways based on activation of Wnt, ERK, or p38 MAP kinase, gene transcription (via β-catenin, the transcription factor NFκB, or by regulation of the maturation of microRNA miR-350) [11,21].

The ENaC channels are also DEG/ENaC family members and are activated in tumor cells by protease cleavage [22]. Due to their similar structure as well as the fundamental similarity of selective filters, the ASIC and ENaC subunits can form heterocomplexes [23]. The interaction between the α-ENaC and ASIC1a subunits, resulting in a cationic current in alveolar cells, has been shown previously in a hybrid system [24]. The heterocomplex formed by the α-ENaC, ASIC1a, and γ-ENaC subunits is characteristic of late-stage glioma cells but not of normal astrocytes [12,13] or lung adenocarcinoma cells [25].

Activated DEG/ENaC channels regulate the important signaling pathways that promote the proliferation and migration of tumor cells of different origins [16,26,27,28,29,30,31], thus targeting these channels is a perspective anti-tumor strategy. For example, inhibitors of the ASIC1a-containing channels—mambalgin-2 (Mamb-2), found in the black mamba (*Dendroaspis polylepis*) venom [32],—suppresses the growth of glioma, leukemia, melanoma, and lung adenocarcinoma cells [25,33,34,35]. Recently, it was demonstrated that Mamb-2 interacts with the tumor-specific heterotrimeric channel composed of the α-ENaC, ASIC1a, and γ-ENaC subunits in melanoma and lung adenocarcinoma cells [25,35]. Moreover, Mamb-2 inhibits the heterotrimeric α-ENaC/ASIC1a/γ-ENaC channel with a higher efficiency than the homotrimeric ASIC1a channel [25]. Although the molecular basis of such a toxin’s selectivity remains unknown.

Here, we studied the Mamb-2 interaction with the homotrimeric ASIC1a and heterotrimeric α-ENaC/ASIC1a/γ-ENaC channels by MD simulations and revealed that the toxin forms additional ionic and hydrophobic contacts with the complementary γ-ENaC(−) subunit. Site-directed mutagenesis of the residues of Mamb-2 and the γ-ENaC(−) subunit from the interacting interface confirmed the discovered mode of the toxin/channel interaction. Our data provide new information about the regulation of acid-sensing ion channels and the demand for rational design of new anti-cancer drugs targeting the tumor-specific α-ENaC/ASIC1a/γ-ENaC heterotrimer.

## 2. Results

Previously, significantly stronger inhibitory activity of Mamb-2 at the α-ENaC/ASIC1a/γ-ENaC heterotrimeric channels in comparison to the ASIC1 homotrimeric channels was reported [25]. Here, we aimed to reveal the molecular and structural basis for such a toxin’s selectivity. To achieve that, we built the models for hetero- and homotrimeric channels in complex with Mamb-2 and ran molecular dynamics (MD) simulations for them in the membrane environment. To prove the initially postulated modeling hypothesis, we performed site-directed mutagenesis and electrophysiology.

### 2.1. Modeling of the Mambalgin-2 Complexes with the ASIC1a and α-ENaC/ASIC1/γ-ENaC Channels

To build a model of the α-ENaC/ASIC1a/γ-ENaC channel, we considered the following points: (1) These channels are assembled counterclockwise; such assembly is provided by inter-subunit complementarity due to a network of hydrogen bonds [36]. Therefore, the α-ENaC, ASIC1a, and γ-ENaC subunits in the heterochannel should also be arranged accordingly. (2) Mamb-2 inhibits currents through the homotrimeric ASIC1a and heterotrimeric α-ENaC/ASIC1a/γ-ENaC channels upon a pH decrease [25]; thus, we proposed that Mamb-2 in complex with the heterotrimeric channel interacts at least with the ASIC1a subunit. (3) According to the cryo-EM structure of the homotrimeric ASIC1a channel with mambalgin-1 (Mamb-1), the closest Mamb-2 homologue (differing by a single substitution Y4F at the N-terminus), mambalgins interact mainly with the ASIC1a subunit located clockwise relative to Mamb-2 (view from the extracellular domain, in the direction of the channel pore), but not with the ASIC1a subunit located counterclockwise [37,38]. We labeled the subunit interacting with Mamb-2 in the hetero- and homocomplexes as the primary (+), and those located counterclockwise as the complementary (−) ones.

**Figure 1:**
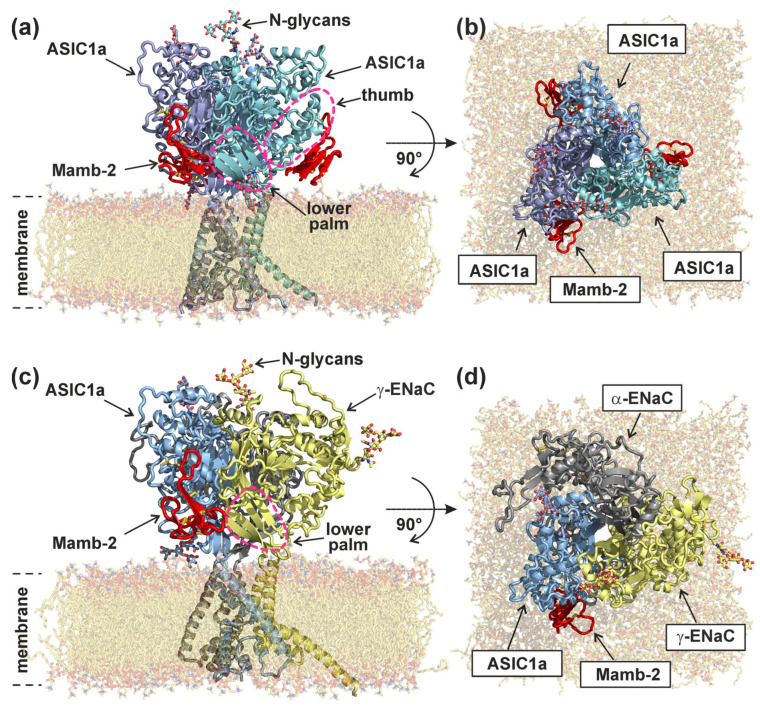
**Models of the complexes of Mamb-2 with ASIC1 homotrimer (a,b) and α-ENaC/ASIC1/γ-ENaC heterotrimer (c,d):** side (**a**,**c**) and top (**b**,**d**) views are shown. Both models are immersed in the phospholipid bilayer and solvated to perform MD calculations; the presented structures result from 500 ns MD.

The models of the hetero- and homomeric channels were built in PyMOL (Figure 1) by superimposition of the individual subunit models on the experimental cryo-EM structure of the homomeric ASIC1a channel in complex with three Mamb-1 molecules whose positions were not altered (PDB code: 7CFT [37]). Structural models of the human ASIC1a (13-470), α-ENaC (50-593), and γ-ENaC subunits (20-579) with Uniprot codes P78348, P37088, and P51170, respectively, were obtained from the AlphaFold database [39,40]. To avoid unwanted artifacts, the disordered N- and C-termini residues were removed from the structures (the final length of the subunits used in the work is shown in the brackets in the previous sentence). The Mamb-2 structure was obtained from the Mamb-1 structure (pdb code 7CFT) by the Y4F substitution using the *mutagenesis* tool in PyMOL.

The models of the Mamb-2 complex with the heterotrimeric α-ENaC/ASIC1a/γ-ENaC and homotrimeric ASIC1a channels were built in a lipid bilayer composed of palmitoyloleoylphosphatidylcholine (POPC) molecules similar to [41] using the CHARMM-GUI [42] Membrane Builder tool [43]. To represent N-glycans at sites 368/395 and 209/497 at the ASIC1a and γ-ENac subunits, respectively, a classic pentasaccharide core composed of two N-Acetylglucosamine (GlcNAc) and three Mannose (Man) residues was used. N-glycans were introduced into the built models of the channels using the Glycan Reader and Modeller tool [44]. The resulting models are shown in Figure 1; further details are provided in Methods.

Molecular dynamics (MD) simulations for both modeled complexes were performed for 500 ns. In both models, Mamb-2 was bound at the adjacent subunits’ interfaces: ASIC1a/γ-ENaC or ASIC1a/ASIC1a (Figure 1). Due to the different number of toxin binding sites, one and three toxin molecules were bound to the heterotrimeric and homotrimeric channels, respectively. Similarly to the cryo-EM structure of ASIC1a/Mamb-1 (PDB code: 7CFT [37]), Mamb-2 interacted mainly with the thumb domain of the ASIC1a(+) subunits and with the lower palm domain of the complementary (−) subunits without contacting the membrane (Figure 1). No considerable contacts of Mamb-2 with N-glycans were observed. The general position of Mamb-2 relative to the subunits and cell membrane in both complexes, as well as the stability of the complexes, did not change during MD.

### 2.2. Mambalgin-2 Interacts More Extensively with the Complementary γ-ENaC(−)-Subunit as Compared with ASIC1(−)

**Figure 2:**
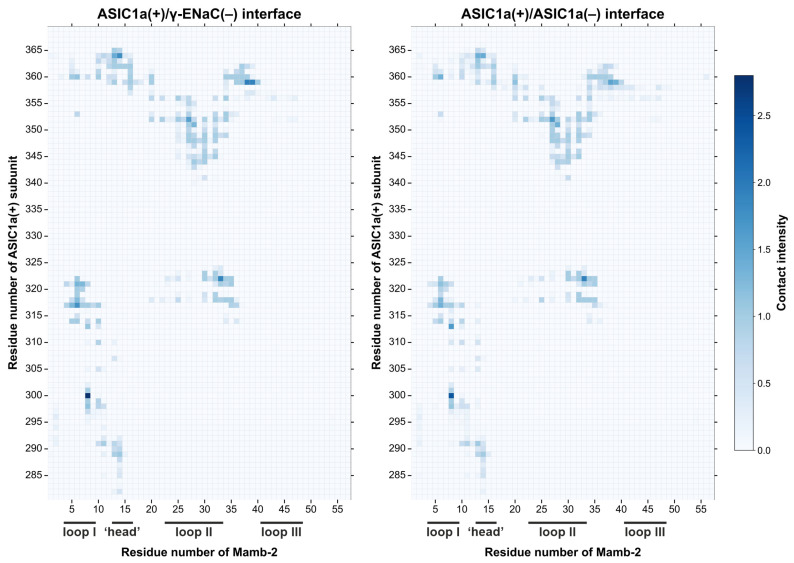
**Contact maps of the Mamb-2 interaction with the primary ASIC1a(+) subunit in heterotrimeric and homotrimeric complexes.** Map cells are colored according to the contact intensity, which corresponds to the summarized lifetime of the contacts of all types between each pair of residues under consideration during the MD simulation of 500 ns. The following types of contacts were considered: ionic bridges, hydrogen bonds, π-cation, stacking, and hydrophobic interactions. ASIC1a residues outside the 280–370 range did not give a significant contribution to the interaction and therefore were not included in the map. The position of the loops’ and ‘head’ regions of Mamb-2 is shown.

**Figure 3:**
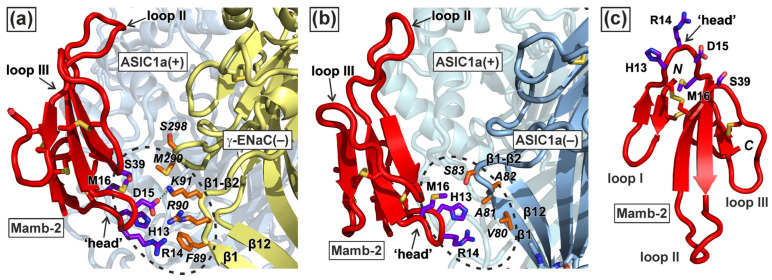
**Amino-acid residues implicated in Mamb-2 interaction with the complementary (−) subunits in the α-ENaC/ASIC1a/γ-ENaC hetero- (a) and ASIC1a homotrimer (b).** Note that interactions with (−)-subunits determine the difference between these two complexes, while the constant part of the Mamb-2/ASIC1a(+) binding interface is not shown for clarity (see also Figure 2). The binding sites Mamb-2/(−)-subunit are shown by dotted lines. The contacting residues from the (−)-subunits are shown in italics. (**c**). Mamb-2 model: loops and ‘head’ are subscribed, along with receptor-interacting residues in the latter.

The MD results with the subsequent intermolecular contact analysis showed that the toxin’s interaction sites at the primary ASIC1a(+) subunit were almost identical in the hetero- and homotrimeric channels. Mamb-2 interacted with the primary ASIC1a(+) subunit in both complexes mainly by the central (II) loop, although some contacts formed by the toxin’s loop I and ‘head’ also were revealed (Figure 2 and Figure 3c, and Tables S1 and S2). Significant differences in the interacting interfaces Mamb-2/channel were observed for the complementary γ-ENaC(−) and ASIC1a(−) subunits of the hetero- and homotrimeric channels, respectively (Figure 3a,b). Mamb-2 interacted with ASIC1a(−) very sparsely (via few hydrophobic contacts), while its contacts with γ-ENaC(−) were more numerous and not limited to just hydrophobic interactions but also included the ionic bridge D15–K91 (Figure 3a,b and Table 1).

In both cases, Mamb-2 is bound to the complementary subunit by just a tip of its “head”, involving mainly hydrophobic interactions. Although the number of such contacts was significantly higher for the α-ENaC/ASIC1a/γ-ENaC channel, this complements the pure hydrophobic interactions with the ionic/hydrogen bond between the toxin’s D15 and the channel’s K91 (Figure 3, Table 1). Notably, in both cases, the toxin’s binding surface was formed by the ‘head’ residues H13–M16 (Figure 3c). No significant staking or π-cation interactions were found in both complexes. An unusual feature arises in the Mamb-2/γ-ENaC interaction (Table 1): the ligand’s R14 interacts (although hydrophobically) with the receptor’s R90, which was weird given the presumed repulsion of these positively charged residues. Indeed, guanidine groups seemed to repel in MD, while aliphatic chains interacted. At the same time, such unusual Arg–Arg short-range interactions, stabilized by water and contributing to intermolecular recognition, were reported previously [45]. We suggest that in the Mamb-2/γ-ENaC interaction, a similar case can be realized.

### 2.3. Interaction with γ-ENaC(−) Determines the High Inhibitory Activity of Mamb-2 at α-ENaC/ASIC1a/γ-ENaC Heterotrimers

**Figure 4:**
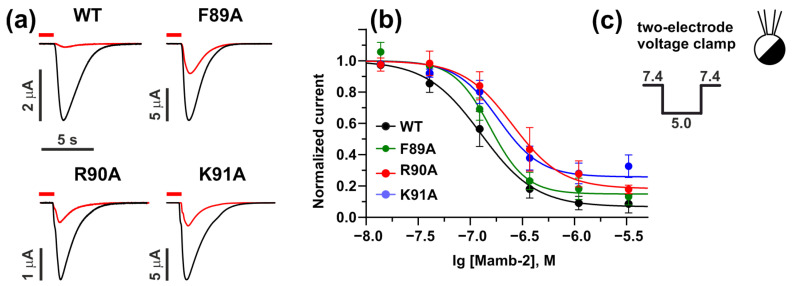
**Mamb-2 inhibitory activity at the α-ENaC/ASIC1a/γ-ENaC channel with mutated γ-ENaC(−) subunit (F89A, R90A and K91A).** (**a**) Representative current traces recorded in *X. laevis* the wild-type (WT) α-ENaC/ASIC1a/γ-ENaC channel or its mutants with substitutions F89A, R90A, and K91A in the γ-ENaC subunit after pH decrease without (black lines) and with (red lines) application of 370 nM of Mamb-2. Oocytes were pre-incubated with Mamb-2 for 15 s (red bar above the trace, the beginning of pre-incubation is off the time scale), and the stimulation phase (pH 5.0) was 7 s. Time and current scale are shown by bars near the traces. (**b**) Dose–response curves for the Mamb-2 inhibitory effect at WT and mutated α-ENaC/ASIC1a/γ-ENaC channels. The response current was normalized to the control experiment for each Mamb-2 concentration. Each data point represents an average from independent experiments in different oocytes ± SEM (*n* = 4–7). The fitted curves are described by Hill’s equation. Parameters describing the curves’ fit are presented in Table 2. (**c**) Protocol of experiments within the two-electrode patch-clamp technique.

To validate the modeling results, we obtained the mutant variants of the γ-ENaC subunit with F89A, R90A, and K91A substitutions. The influence of these mutations on the activity of Mamb-2 at the α-ENaC/ASIC1a/γ-ENaC channel was analyzed in electrophysiological experiments in *X. laevis* oocytes, which express wild-type (WT) and mutant variants of the channel. Notably, the introduced mutations didn’t affect the mean expression level of the channel, as was shown by the comparison of peak current distributions (see Figure S1). We found that 370 nM of Mamb-2 almost completely inhibited the current through the WT α-ENaC/ASIC1a/γ-ENaC heterotrimer. However, in the case of the α-ENaC/ASIC1a/γ-ENaC mutants, Mamb-2 demonstrated lower activity (Figure 4a).

The analysis of “dose-response” curves showed that the mutations R90A and K91A in the γ-ENaC subunit significantly diminished the activity of Mamb-2 at the α-ENaC/ASIC1a/γ-ENaC heterotrimeric channel (Figure 4b, Table 2). The curves for R90A and K91A channel mutants moved right regarding the curve for the WT channel, demonstrating a significant increase in IC_50_ from ~130 nM of the WT heterotrimeric channel to ~250 nM and 190 nM, respectively. Simultaneously, the changes in the bottom of the curves (the value of maximal inhibitory response) for the R90A and K91A mutants of the α-ENaC/ASIC1a/γ-ENaC channel were observed, pointing to possible allosteric regulation by Mamb-2.

### 2.4. Mamb-2 “Head” Mutations Diminish Its Inhibitory Activity at the α-ENaC/ASIC1a/γ-ENaC Channel but Not at ASIC1a Channel

To prove the participation of the Mamb-2 ‘head’ in the interaction with the complementary γ-ENaC subunit revealed by the MD study (Figure 2, Table 1) and the greater role of this interaction in α-ENaC/ASIC1a/γ-ENaC as compared with the ASIC1a channel, we produced the toxin mutants with substitutions H13A, R14A, D15A, and R14A/D15A. Mutations did not alter the overall Mamb-2 structure, according to 1D ^1^H-NMR analysis. The NMR spectra of the mutants were very similar to the spectrum of WT Mamb-2, suggesting that mutant toxins’ structures did not change (Figure S2).

**Figure 5:**
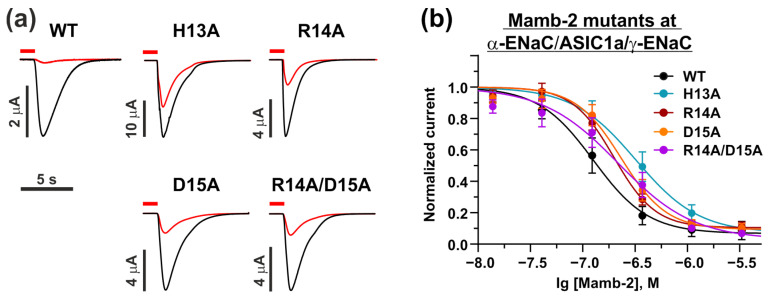
**Inhibitory activity of Mamb-2 and its mutants H13A, R14A, D15A, and R14A/D15A at the α-ENaC/ASIC1a/γ-ENaC channel.** (**a**) Representative traces of currents recorded in *X. laevis* oocytes, which express WT α-ENaC/ASIC1a/γ-ENaC channel after pH decrease without (black lines) and with (red lines) application of 370 nM of Mamb-2 and its mutants. Oocytes were pre-incubated with Mamb-2 for 15 s (red bar above the trace, the beginning of pre-incubation is off the time scale), and the pH drop phase (pH 5.0) was 7 s. Time and current scale are shown by bars near the traces. (**b**) Dose–response curves for the Mamb-2 and its mutants at the α-ENaC/ASIC1a/γ-ENaC channel. The response current was normalized to the control experiment for each Mamb-2 concentration. Each data point represents an average from independent experiments in different oocytes ±SEM (*n* = 4–7). The fitted curves are described by Hill’s equation. Parameters describing the curves’ fit are presented in Table 3.

We found that no mutant toxins were able to fully inhibit the currents through the WT α-ENaC/ASIC1a/γ-ENaC channel at the concentration of 370 nM, while WT Mamb-2 (Figure 5a). For each Mamb-2 mutant, we obtained “dose–response” curves and compared them to the curve of the WT toxin. It appeared that every single mutation resulted in significantly diminished toxin activity at the α-ENaC/ASIC1a/γ-ENaC heterochannel (Figure 5b). IC_50_ for the mutants H13A, R14A, D15A, and R14A/D15A became ~350, 200, 230, and 220 nM, respectively, while the IC_50_ of WT Mamb-2 was found to be ~130 nM. Notably, the diminished activity of the double mutant R14A/D15A was obvious but didn’t reach statistical significance (Table 3). No significant changes in the bottom of the curves for any of the Mamb-2 mutants were revealed (Table 3).

**Figure 6:**
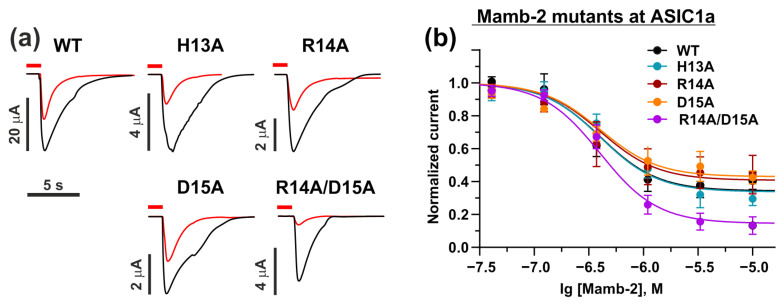
**Inhibitory activity of Mamb-2 and its mutants H13A, R14A, D15A, and R14A/D15A at the ASIC1a channel.** (**a**) Representative traces of currents recorded in *X. laevis* oocytes, which express WT ASIC1a channel after pH decrease without (black lines) and with (red lines) application of 1.1 µM of Mamb-2 and its mutants. Oocytes were pre-incubated with Mamb-2 for 15 s (red bar above the trace, the beginning of pre-incubation is off the time scale), and the pH drop phase (pH 5.0) was 7 s. Time and current scale are shown by bars near the traces. (**b**) Dose–response curves for the Mamb-2 and its mutants at the ASIC1a channel. The response current was normalized to the control experiment for each Mamb-2 concentration. Each data point represents an average from independent experiments in different oocytes ±SEM (*n* = 4–7). The fitted curves are described by the single-component Hill’s equation. Parameters describing the curves’ fit are presented in Table 4.

At the same time, the ’head’ mutations H13A, R14A, and D15A caused no influence on the Mamb-2 activity at the ASIC1a homotrimeric channel (Figure 6a,b). In contrast to the situation with the α-ENaC/ASIC1a/γ-ENaC channel, the double mutation R14A/D15A and single mutation H13A resulted in the increased toxin’s activity at the ASIC1a channel with significantly enhanced maximal inhibitory response, although without significant change in IC_50_ (Figure 6a,b, Table 4). Thus, Mamb-2 WT and its mutants H13A, R14A, and D15A demonstrated only partial inhibitory activity at the ASIC1a channel even at high concentrations (10 µM), while the double mutation R14A/D15A resulted in nearly full inhibition of the ASIC1a channel at this concentration (Figure 6b, Table 4).

## 3. Discussion

Acid-sensing trimeric ion channels of the DEG/ENaC family are activated by external acidification (pH drop) and are important regulators of different essential processes such as mechanosensitivity, pain perception, synaptic plasticity, anxiety response, cancer progression, etc., [10,46]. The composition of the channel’s trimers determines its sensitivity to the pH drop or other physiological stimuli and shapes the adequate response to an external signal [11]. The formation of channels with altered composition and response patterns in comparison to physiologically normal channels may serve as a mechanism of adaptation of the cells to different pathological conditions. For example, during cancer progression, the formation of the tumor-specific heterotrimeric α-ENaC/ASIC1a/γ-ENaC channels was shown [12,13]. The activation of such channels may drive the adaptation of tumor cells to external acidification and activate different mitogenic signaling pathways [10]. Thus, targeting such cancer-specific heterotrimeric channels may be a perspective strategy for the treatment of different oncopathologies.

Previously, it was shown that mambalgin-2, the three-finger protein (TFP, [47]) from the *Mamba* venom initially described as the inhibitor of the ASIC1a channels [48], interacts with the heterotrimeric α-ENaC/ASIC1a/γ-ENaC channels in various cancer cells, and this interaction results in control of the homeostasis of cancer cells [25,35]. Moreover, mambalgin-2 inhibits currents evoked by the pH drop at the α-ENaC/ASIC1a/γ-ENaC channels with higher efficiency than at the ASIC1a channels (Table 3 and Table 4, and [25]). Here, we aimed to elucidate the molecular basis underlying the mambalgin-2 selectivity regarding the heterotrimeric α-ENaC/ASIC1a/γ-ENaC channels.

We performed computer modeling of the interaction of mambalgin-2 with two types of channels: heterotrimeric α-ENaC/ASIC1a/γ-ENaC and homotrimeric ASIC1a. We found that the toxin forms a similar interaction pattern with the primary ASIC1a(+) subunit in both cases, mostly by the central (II) loop (Figure 2), which agrees well with the previous mutagenesis data [48]. However, the interaction mode of the toxin with the complementary (−) subunits was different. Almost all residues forming the interaction interface Mamb-2/γ-ENaC(−) in complex with the heterotrimeric channel were located in the ‘head’ of the toxin’s molecule (Figure 3a). The ‘head’ region of Mamb-2 formed the set of hydrophobic and ionic contacts with the γ-ENaC(−) subunit, while the number of the ‘head’ contacts with the ASIC1a(−) subunit in complex with the homotrimeric channel was obviously lower (Figure 3a,b and Table 1, Tables S1 and S2). Site-directed mutagenesis with the subsequent electrophysiological studies confirmed the importance of the ‘head’ residues for the toxin’s interaction just with the α-ENaC/ASIC1a/γ-ENaC channels, but not with the ASIC1a channels. Thus, the stronger activity of Mamb-2 at the α-ENaC/ASIC1a/γ-ENaC channels can be explained by the existence of additional contacts of the toxin’s ‘head’ with the complementary γ-ENaC(−) subunit.

The main functional epitopes of TFPs are located in the disordered loops I-III [49,50,51,52]. The ‘Head’ region of TFPs also has a disordered structure (Figure 3c) and can be considered an auxiliary binding site regulating TFP’s interaction with other supplementary partners necessary for the function of the ‘main’ receptor. These supplementary partners can be either the cell membrane surrounding the nicotinic acetylcholine receptor [53,54] or other receptors, for example, receptor-tyrosine kinases in the case of nicotinic acetylcholine receptors and the human SLURP-1 protein in cancer cells [51]. Here, on the example of mambalgin-2, we revealed an additional ‘application’ of the ‘head’ region of TFPs: the regulation of the interaction selectivity between various types of acid-sensing channels. Probably, the ‘head’ moiety can be used for transplanting one or another activity to TFPs and to drug design with directed specificity.

Surprisingly, wild-type Mamb-2 fully inhibited the α-ENaC/ASIC1a/γ-ENaC channels and only partially inhibited the ASIC1a ones (Figure 4 and Figure 5), pointing to possible allosteric modulation of the homotrimeric channel by the toxin. Mutations R90A and K91A of the heterotrimeric channel led to increased IC_50_ (from 130 ± 20 to 250 ± 60 and 190 ± 30 nM, respectively) and the diminished maximal inhibitory activity of Mamb-2 (from 100% to ~70%, Figure 4, Table 2), indicating the appearance of the allosteric effects with switching of the pharmacology of the α-ENaC/ASIC1a/γ-ENaC channel to the properties of the homotrimeric ASIC1a channel. On the other hand, the introduction of the double mutation R14A/D15A into the Mamb-2 molecule also changed the mode of the toxin interaction with the homotrimeric channel in the direction of the heterotrimeric channel (increase in the maximal inhibitory activity of Mamb-2 from ~60% to ~85% with slight decrease in IC_50_, Figure 6, Table 4). Thus, the changes in the binding interface both from the toxin’s and channel’s sides could shift the pharmacology of heterotrimeric and homotrimeric acid-sensing channels to each other.

Previously, indirect data indicating that Mamb-2 inhibits and stabilizes the desensitization state of the channels containing the ASIC1a subunit in cancer cells were obtained [33,34]. Here, we built the models of the complexes of Mamb-2 with the α-ENaC/ASIC1a/γ-ENaC and ASIC1a channels, and these complexes were stable at MD during the 500 ns trajectory. According to these models, Mamb-2 interacts with the α4 and α5 helices of the thumb domain of the primary ASIC1a(+) subunit (Figure 1), which has an allosteric effect on the acid (proton-binding) pocket, keeping it in an expanded state with a simultaneously closed ion channel [38]. This channel’s state is characterized by a noticeable weakening of pH-sensing and a decrease in the current through the channel [38], which is in line with our present data (Figure 4, Figure 5 and Figure 6). On the other hand, Mamb-2 interacts with the residues from the β1-β2 linker of the palm domain of the complementary (−) subunits (F89, R90, K91 in the case of the γ-ENaC (−) subunit and V80, A81, A82, and S83 in the case of ASIC1a (−) subunit). Previously, in the example of the homotrimeric ASIC1a channel, the important role of reorientation of the β1-β2 linker in switching the channel from the low-pH open state to the low-pH desensitized state was shown [55]. This means that the interaction of Mamb-2 with the β1-β2 linker can stabilize either the open or desensitized states of the channels. Keeping in mind that no activation effect from Mamb-2 was observed (Figure 4, Figure 5 and Figure 6), this indicates that the toxin stabilizes the desensitization state of the α-ENaC/ASIC1a/γ-ENaC and ASIC1a channels.

The function of the α-ENaC/β-ENaC/γ-ENaC channels can be regulated by different molecules and factors such as extracellular Na^+^, Cl^−^, H+, metals, proteases, amiloride, and sheer stress [56]. Presently, several extracellular regulatory sites in the heterotrimeric channel were described: the extracellular sites located at the finger domains of the α-ENaC and γ-ENaC subunits, which can affect Na^+^ self-inhibition [22,57], and the amiloride binding pore domain [58]. Moreover, cross-linking of the palm domains of α-ENaC or β-ENaC with the thumb domains of γ-ENaC or α-ENaC, respectively, resulted in strong inhibition of the α-ENaC/β-ENaC/γ-ENaC channel activity [56]. Here, we substituted the β-ENaC subunit with the ASIC1a subunit and showed that linking the thumb domain of ASIC1a with the palm domain of γ-ENaC within the α-ENaC/ASIC1a/γ-ENaC channel by the Mamb-2 molecule (Figure 3a) also resulted in the channel inhibition (Figure 4). Thus, a new mode of regulation of the heterotrimeric ASIC1a/ENaC channels was revealed.

## 4. Conclusions

Thus, by computer modeling, we predicted the difference in the binding modes of mambalgin-2 with the heterotrimeric α-ENaC/ASIC1a/γ-ENaC and homotrimeric ASIC1a channels. The importance of the ‘head’ region of the toxin for the interaction with the heterotrimeric channel was confirmed by site-directed mutagenesis and electrophysiology. A new mode of allosteric regulation of ENaC channels was revealed. The data obtained provide new insights into the regulation of various types of acid-sensing ion channels and the development of new strategies for cancer treatment.

## 5. Materials and Methods

### 5.1. Computer Modeling Methods

#### 5.1.1. Molecular Dynamics Calculations

MD trajectories for the heterotrimeric α-ENaC/ASIC1a/γ-ENaC and homotrimeric ASIC1a channels in complex with Mamb-2 were calculated in the all-atom CHARMM36m forcefield [59] and TIP3P water model via GROMACS [60]. For a more accurate description of π-cation interactions, an additional set of CHARMM36-WYF parameters [61] was used. MD calculations included the following stages: energy minimization (steepest descent algorithm), NVT relaxation (250 ps), NPT relaxation with C-rescale barostat [62] (2000 ps), and production MD (500 ns).

The MD calculations for an equilibrated system were performed in an NPT ensemble at a temperature of 310 K with a V-rescale thermostat [63] and a Parinello-Raman barostat [64] with a time step of 2 fs. No position restraints were applied to any molecules during the production MD phase, so the structures of the proteins and N-glycans were allowed to relax and change.

#### 5.1.2. Contacts Analysis

For analysis of intermolecular contacts between Mamb-2 and the heterotrimeric α-ENaC/ASIC1a/γ-ENaC and homotrimeric ASIC1a channels, we utilized our in-house IMPULSE software package (cont_stat.js procedure) [65]. Ionic, ion-dipole, π-cationic, staking interactions, hydrogen bonds, and hydrophobic contacts were analyzed. Hydrophobic contacts were determined according to the concept of molecular hydrophobic potential (MHP) [66]. In the case of the homotrimeric ASIC1a channel data, the Mamb-2 interaction lifetime was averaged between three toxin molecules.

A complete list of all found contacts is available in Tables S1 and S2 for the hetero- and homotrimer channels, respectively. The values in the tables are the relative lifetimes of interactions between the Mamb-2 and channels’ primary (+) and complementary (−) subunit residues; one can analyze them using the sorting and filtering tools of the table processing program. Relative lifetime is the fraction of MD time when the contact is observed, from absence (0) to presence throughout the whole trajectory (1).

Using the obtained data on the contacts lifetimes from the Impulse software package, we built maps of the interaction intensity of Mamb-2 with the primary ASIC1a(+) subunit in the ASIC1a(+)/ASIC1a(+) and ASIC1a(+)/γ-ENaC(−) interfaces in the homotrimeric and heterotrimeric channels, respectively. To do that, we performed a summation of all the interaction lifetimes of different types of contacts between each residue of Mamb-2 and each residue of ASIC1a(+) in both complexes. The data on the contacts lifetime used for summation are represented in Tables S1 and S2, as are the initial *csv* files obtained from the Impulse software package after MD trajectory analysis. The summation of lifetimes was performed using our own Python script. The following types of contacts lifetimes were summarized: ionic bridges, hydrogen bonds, π-cation, stacking, and hydrophobic interactions. The resulting heatmaps were prepared *via* Matplotlib and Seaborn library utilities. Developed scripts for data analysis and contacts maps preparation are available at the Zenodo repository.

The following software versions were used: GROMACS 2022.4; CHARMM-GUI 3.8; IMPULSE 21.09; and PyMOL 2.5.5.

### 5.2. Recombinant Mambalgin-2 and Its Mutants Production

Recombinant Mamb-2 and its mutant variants with the H13A, R14A, D15A, and R14A/D15A substitutions were produced in *E. coli* as described previously [25]. The homogeneity and purity of the mambalgin-2 were confirmed by SDS-PAGE, HPLC, and MALDI-MS in the reaction, and Ellman’s reagent (Sigma-Aldrich, St. Louis, MO, USA) was used to confirm the disulfide bond formation. The proper spatial structure was confirmed by 1D ^1^H NMR.

### 5.3. Electrophysiological Recordings in X. laevis Oocytes

For expression of the human homotrimeric ASIC1a, heterotrimeric α-ENaC/ASIC1a/γ-ENaC, or heteromeric channel with F89A, R90A, and K91A mutations in the γ-ENAC subunit, Xenopus laevis oocytes were harvested and injected with synthesized mRNA coding the corresponding genes as described in [67]. Two-electrode voltage-clamp recordings were done using the TEC-03X amplifier (npi electronic GmbH, Tamm, Germany) at a holding potential of −50 mV. The output signal was filtered at 20 Hz by a four-pole Bessel filter and digitized at 1 kHz using the National Instruments BNC-6251 card, controlled by WinWCP 5.2.7. Microelectrodes were fabricated with 1.0–1.5 MOhm resistance and filled with 3 M KCl. The external perfusion solution for oocytes was ND-96 medium (96 mM NaCl, 2 mM KCl, 1.8 mM CaCl_2_, 1 mM MgCl_2_, 10 mM HEPES, pH 7.4) for both ASIC1a and α-ENaC/ASIC1a/γ-ENaC, and it was fed to a perfusion chamber (volume ~50 µL) using a gravity-flow system at speed 2.5 mL/min. The currents were stimulated by a pH drop to 5.0 for both ASIC1a and α-ENaC/ASIC1a/γ-ENaC.

The pH drop was performed by fast perfusion solution exchange for 0.1 s to ND-96 medium with 10 mM MES instead of HEPES, adjusted to the target pH, and kept for 7 s; after that, the solution was exchanged to ND-96 with a pH of 7.4. The treatment of oocytes with the Mamb-2-containing solution was performed by solution exchange in the recording chamber for 15 s before the current stimulation. Lyophilized Mamb-2 was dissolved in 100% DMSO in a 10-mM stock solution and then diluted to a target concentration with ND-96 medium before the experiment. Amiloride (Sigma-Aldrich) was diluted in ND-96 medium (pH 7.4) from the 500-mM stock in 100% DMSO. The DMSO concentration in the recording solution did not exceed 1%. The exchange of extracellular solutions was performed using a computer-controlled valve system.

Mamb-2 concentrations for the dose–response curves were 0–10 μM. Each oocyte was tested with all concentrations of Mamb-2. Dose–response curves were fitted with the Hill equation: Y = 1/(1 + 10^((LogIC50 − X)×nH)^), where Y is the normalized current amplitude (independent normalization for each oocyte), X is the lg of concentration of the ligand concentration, and nH is the Hill coefficient (slope factor). Fit parameters were compared with one-way ANOVA and Dunnett’s multiple comparison test; for IC_50,_ the comparison of LogIC_50_ was done instead of IC_50_ itself.

### 5.4. Statistical Analysis

Data are presented as mean ± SEM. The number of samples (*n*) is shown in the figure legends. The absence of outliers in each dataset was confirmed by the Grubbs’ test (alpha = 0.05). The data were tested for normality (Shapiro–Wilk test, at *p* = 0.05) and for the homogeneity of variances (Levene’s test, at *p* = 0.05). The data were analyzed using the F-test, as indicated in the figure legends. Differences in the data were considered statistically significant at *p* < 0.05. Analysis was performed using the GraphPad Prism 8.0 software (GraphPad Software).

## Figures and Tables

**Table 1:** **Mambalgin-2 contacts with the complementary γ-ENaC(–) and ASIC1(–) subunits found in MD.** The interaction type is denoted in the brackets: hydrogen bond (H), ionic bridge or ion–dipole interaction (I), and hydrophobic contact (M) (according to “Molecular Hydrophobic Potential”). H and I contacts with a lifetime >10% (of 500 ns MD trajectory) and M contacts with a lifetime >30% are listed. Hydrophobic (M) contacts between apparently polar/charged residues denote the interaction of non-polar fragments of these residues.

Mambalgin-2	γ-ENaC(–)	ASIC1a(–)
**H13**	F89 (M)	A81 (M)
	R90 (M)	
	K91 (M)	
**R14**	F89 (M)	V80 (M)
	R90 (M)	A81 (M)
	K91 (M)	
**D15**	K91 (I, H, M)	
**M16**	K91 (M)	A81 (M)
	S298 (M)	A82 (M)
	M299 (M)	S83 (M)
**S39**	K91 (M)	

**Table 2:** **Parameters of Mamb-2 inhibitory activity at the α-ENaC/ASIC1a/γ-ENaC channel with the mutated γ-ENAC subunit.** ‘Bottom’ is the value of maximal inhibitory response, nH—the Hill coefficient, IC_50_—concentration of the ligand at which the amplitude of the current through the channel was inhibited by 50%. ** (*p* < 0.01) and **** (*p* < 0.0001) indicate significant differences between the data groups and the wild-type group (WT) by one-way ANOVA with Dunnett post-hoc test, n.s. (*p* > 0.05) indicates the absence of statistical difference.

γ-ENaC	IC_50_, nM	Bottom	nH
**WT**	130 ± 20	0.07 ± 0.04	1.6 ± 0.4
**F89A**	150 ± 20 ^n.s.^	0.14 ± 0.03 ^n.s.^	2.5 ± 0.7 ^n.s.^
**R90A**	250 ± 60 ****	0.18 ± 0.07 ^n.s.^	1.8 ± 0.6 ^n.s.^
**K91A**	190 ± 30 **	0.26 ± 0.05 ^n.s.^	2.1 ± 0.6 ^n.s.^

**Table 3:** **Parameters of inhibitory activity of the Mamb-2 mutants at the α-ENaC/ASIC1a/γ-ENaC channel.** ‘Bottom’ is the value of the maximal inhibitory response, nH—the Hill coefficient, IC_50_—concentration of the ligand at which the amplitude of the current through the channel was inhibited by 50%. * (*p* < 0.05), **** (*p* < 0.0001) indicates significant differences between the data groups and the wild-type group (WT) by one-way ANOVA with Dunnett post-hoc test, n.s. (*p* > 0.05) indicates the absence of statistical difference.

Mambalgin-2 Mutation	IC_50,_ nM	Bottom	nH
**WT**	130 ± 20	0.07 ± 0.04	−1.6 ± 0.4
**H13A**	350 ± 60 ****	0.07 ± 0.05 ^n.s.^	−1.4 ± 0.3 ^n.s.^
**R14A**	200 ± 20 ****	0.10 ± 0.04 ^n.s.^	−2.2 ± 0.3 *
**D15A**	230 ± 30 ****	0.09 ± 0.05 ^n.s.^	−2.0 ± 0.4 ^n.s.^
**R14A/D15A**	220 ± 40 ****	0.03 ± 0.08 ^n.s.^	−1.2 ± 0.2 ^n.s.^

**Table 4:** **Parameters of inhibitory activity of the Mamb-2 mutants at the ASIC1a channel.** ‘Bottom’ is the value of maximal inhibition, nH—the Hill coefficient, IC_50_—concentration of the ligand at which the amplitude of the current through the channel was inhibited by 50%. * (*p* < 0.05), ** (*p* < 0.01), and **** (*p* < 0.0001) indicate significant differences between the data groups and the wild-type group (WT) by one-way ANOVA with Dunnett post-hoc test, n.s. (*p* > 0.05) indicates the absence of statistical difference.

Mambalgin-2 Mutation	IC_50,_ nM	Bottom	nH
**WT**	310 ± 60	0.39 ± 0.04	2.9 ± 1.5
**H13A**	510 ± 1500 ^n.s.^	0.28 ± 0.05 **	1.5 ± 0.4 *
**R14A**	250 ± 300 ^n.s.^	0.44 ± 0.07 ^n.s.^	1.7 ± 0.9 ^n.s.^
**D15A**	300 ± 400 ^n.s.^	0.42 ± 0.06 ^n.s.^	1.0 ± 0.35 **
**R14A/D15A**	470 ± 70 ^n.s.^	0.13 ± 0.05 ****	1.9 ± 0.4 ^n.s.^

## Data Availability

The data presented in this study are available in supplementary material.

## Supplementary Materials

The following supporting information can be downloaded at https://www.mdpi.com/article/10.3390/toxins15100612/s1, Figure S1. Scatter plot of peak current amplitudes for α-ENaC/ASIC1a/γ-ENaC oocytes with WT, F89A, R90A, K91A γ-ENaC subunits. No statistically significant differences between means were detected by one-way ANOVA (F(3, 21) = 0.4985, *p* = 0.6873), Figure S2. Amide regions of ^1^H 1D NMR spectra of Mamb-2 variants (700 MHz, 30 °C, pH 4.0); Supplementary Tables S1 and S2 for contacts lifetime data, MD trajectories, Python scripts and CSV data can be downloaded at Zenodo repository https://doi.org/10.5281/zenodo.8303194 (accessed on 8 October 2023).
