# Supplementary material for: Molecular Basis for Mambalgin-2 Interaction with Heterotrimeric α-ENaC/ASIC1a/γ-ENaC Channels in Cancer Cells
Source: Toxins (Basel). 2023 Oct 13;15(10):612. doi: 10.3390/toxins15100612 (PMC10610865; doi:10.3390/toxins15100612)
Supplement: Supplementary file 1 [file toxins-15-00612-s001.zip › toxins-2617299-supplementary.pdf]

toxins

Supplementary material

Molecular Basis for Mambalgin-2 Interaction with Heterotrimeric α-ENaC/ASIC1a/γ-ENaC Channels in Cancer Cells

Ekaterina N. Lyukmanova [1,2,3,4,*,†], Maxim M. Zaigraev [2,3,†], Dmitrii S. Kulbatskii [2,†], Aizek B. Isaev [2,3], Ilya D. Kukushkin [2,3], Maxim L. Bychkov [2], Mikhail A. Shulepko [1], Anton O. Chugunov [2,3] and Mikhail P. Kirpichnikov [2,4]

1 Faculty of Biology, MSU-BIT Shenzhen University, Shenzhen 518172, China; mikhailshulepko@yandex.ru
2 Shemyakin-Ovchinnikov Institute of Bioorganic Chemistry, Russian Academy of Sciences, Miklukho-Maklaya 16/10, Moscow 117997, Russia; maximzaigraev@yandex.ru (M.M.Z.); d.kulbatskiy@gmail.com (D.S.K.); isaev.aizek@gmail.com (A.B.I.); ilya23kuku@gmail.com (I.D.K.); maksim.bychkov@gmail.com (M.L.B.); batch2k@yandex.ru (A.O.C.); kirpichnikov@inbox.ru (M.P.K.)
3 Phystech School of Biological and Medical Physics, Moscow Institute of Physics and Technology (National Research University), Institutsky Lane 9, Dolgoprudny, Moscow 141701, Russia
4 Interdisciplinary Scientific and Educational School of Moscow University «Molecular Technologies of the Living Systems and Synthetic Biology», Faculty of Biology, Lomonosov Moscow State University, Leninskie Gory, Moscow 119234, Russia
* Correspondence: lyukmanova_ekaterina@smbu.edu.cn
† These authors contributed equally to this work.

Citation: Lyukmanova, E.N.; Zaigraev, M.; Kulbatskii, D.; Isaev, I.; Kukushkin, I.; Bychkov, M.; Shulepko, M.; Chugunov, A.; Kirpichnikov, M. Molecular Basis for Mambalgin-2 Interaction with Heterotrimeric α-ENaC/ASIC1a/γ-ENaC Channels in Cancer Cells. *Toxins* **2023**, *15*, 612. https://doi.org/10.3390/toxins15100612

Received: 8 September 2023
Revised: 30 September 2023
Accepted: 7 October 2023
Published: 13 October 2023

Copyright: © 2023 by the authors. Licensee MDPI, Basel, Switzerland. This article is an open access article distributed under the terms and conditions of the Creative Commons Attribution (CC BY) license (https://creativecommons.org/licenses/by/4.0/).

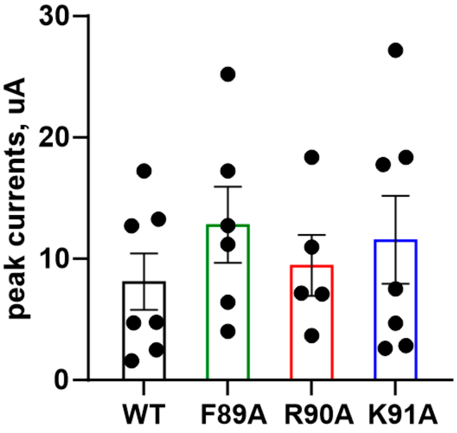

Figure S1. Scatter plot of peak current amplitudes for α-ENaC/ASIC1a/γ-ENaC oocytes with WT, F89A, R90A, K91A γ-ENaC subunits. No statistically significant differences between means were detected by one-way ANOVA (F(3, 21) = 0,4985, p=0,6873).

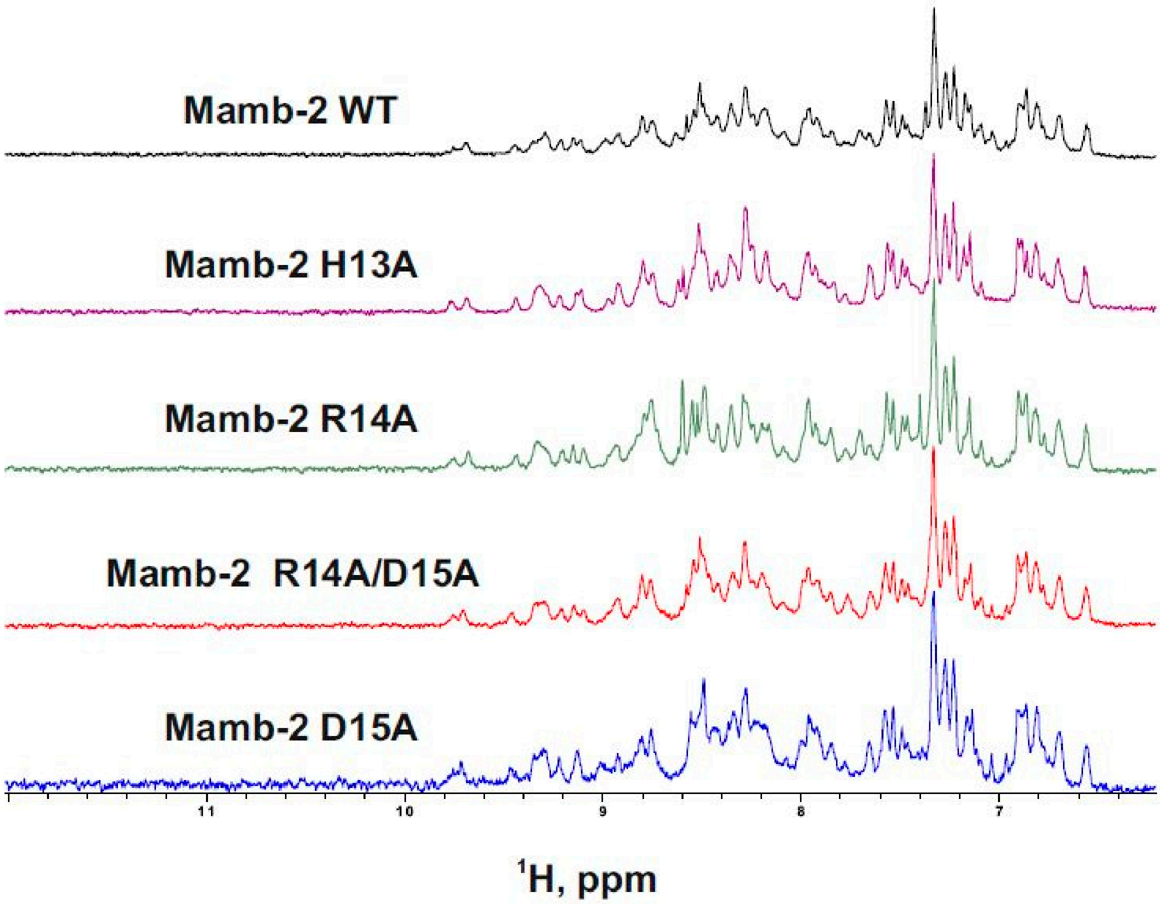

Figure S2. Amide regions of ^{1}H 1D NMR spectra of Mamb-2 variants (700 MHz, 30℃, pH 4.0).
